# Fiber-bundle illumination: realizing high-degree time-multiplexed multifocal multiphoton microscopy with simplicity

**DOI:** 10.1038/s41598-018-33286-1

**Published:** 2018-10-05

**Authors:** Jiun-Yann Yu, Sunduck Kim, Young Bo Shim, Daniel B. Holland, Marco A. Allodi, Chao-Yuan Yeh, Geoffrey A. Blake, Young-Geun Han, Chin-Lin Guo

**Affiliations:** 1 Division of Biology and Biological Engineering, California Institute of Technology, Pasadena, CA 91125 USA; 2 Department of Physics, Hanyang University, Seoul, 133-791 South Korea; 3 Division of Chemistry and Chemical Engineering, California Institute of Technology, Pasadena, CA 91125 USA; 4 Department of Pathology, University of Southern California, Los Angeles, CA 90033 USA; 5 Division of Geological and Planetary Sciences, California Institute of Technology, Pasadena, CA 91125 USA; 6 Institute of Physics, Academia Sinica, NanKang District, Taipei, Taiwan 11579 Republic of China

## Abstract

High-degree time-multiplexed multifocal multiphoton microscopy was expected to provide a facile path to scanningless optical-sectioning and the fast imaging of dynamic three-dimensional biological systems. However, physical constraints on typical time multiplexing devices, arising from diffraction in the free-space propagation of light waves, lead to significant manufacturing difficulties and have prevented the experimental realization of high-degree time multiplexing. To resolve this issue, we have developed a novel method using optical fiber bundles of various lengths to confine the diffraction of propagating light waves and to create a time multiplexing effect. Through this method, we experimentally demonstrate the highest degree of time multiplexing ever achieved in multifocal multiphoton microscopy (~50 times larger than conventional approaches), and hence the potential of using simply-manufactured devices for scanningless optical sectioning of biological systems.

## Introduction

The ability to perform high-frame-rate, three-dimensional, quantitative imaging of biological systems is playing an ever more important role in modern life science research. Among many under-development approaches, wide-field optical-sectioning fluorescence microscopy has displayed unique advantages over conventional single-focus laser scanning microscopy. The currently available methods for wide-field optical-sectioning, however, possess various complexities and limitations. Selective plane illumination microscopy (SPIM), for example, provides detailed information on cell motion in embryonic development^1,2^, but its illumination mechanism leads to tradeoffs between the size of the field of view and the axial resolution. The close proximity of separate illumination and imaging optics further increases the complexity of SPIM, leading to significant difficulties in sample handling. Other examples of limitations in present techniques include the difficulty of multi-wavelength excitation in temporal focusing microscopy^3^ and the reduced signal-to-noise ratio in structured illumination microscopy^4^.

In comparison with the aforementioned methods, an appealing approach to wide-field optical-sectioning is to create a high degree of time multiplexing among the foci in multifocal multiphoton fluorescence microscopy^5^, thereby producing a high spatial density of foci without increasing out-of-focus excitation caused by interfocal interference. To achieve a scanningless effect, it was estimated that ~300 distinct time-delay steps (hereafter referred to as unique time delays) are needed for multifocal multiphoton microscopy^6^. Conventional time-multiplexing devices, however, can provide only upto three unique time delays, due to the difficulty in their manufacturing processes^7^. In the conventional time-multiplexing device, temporal separations are created among the light pulses propagating through an array of glass pillars of various heights^5,7^. Due to the diffraction associated with free-space propagation, these light pulses diverge at a certain distance after exiting the pillars, even with a perfect collimation before entering the pillars. Such a divergence can cause a leakage of light between neighboring pillars, thereby degrading temporal separation and optical sectioning. The leakage, nevertheless, can be reduced by adjusting the cross-sectional area of pillars, *A*, with the height differences, Δ*h*, as demonstrated in our previous work, where we showed that having a negligible light leakage requires:

1 $$A\ge {\lambda }_{0}\,{\rm{\Delta }}{h}_{{\rm{\max }}}.$$

Here, *λ*_0_ is the central wavelength of the light pulse, *A* is the cross-sectional area of a single pillar, and Δ*h*_*max*_ is the maximal height difference among the pillars^6^. To achieve a scanningless wide-field imaging effect, Eqn. (1) provides an estimate for the dimensions of a time-multiplexing device. With an ~100-fs duration ultrafast pulse train, the device needs Δ*h*_*max*_ > 30 mm and *A* (its entire cross-sectional area) >100 cm^2^ ^6^. Fulfilling both requirements on one single optical device is far beyond the current fabrication capability for optical-quality manufacturing. Alternatively, light leakage can be avoided by aligning the ends of the pillars on a single plane perpendicular to the optical axis of the imaging system (as seen in a conventional microlens array), but such a device would provide no time multiplexing.

**Figure 1:**
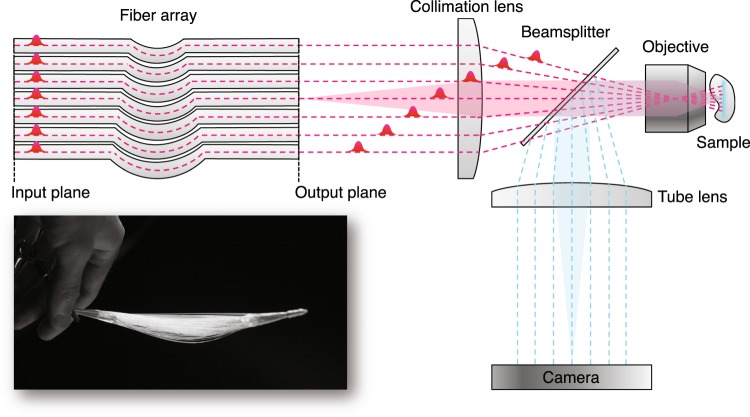
Schematic of the optical system. After a light pulse passes through the fiber bundle, multiple light pulses are generated with spatial and temporal separations, and create a plane of foci at the focal plane of the microscope objective. Because of the temporal separation, the optical properties of each focus can be considered identical to those of the conventional single-focus multiphoton microscopy^5^. The fluorescence signals emitted within the excited plane are then collected by the microscope objective and routed to a camera for wide-field imaging^17^. The dashed lines show the central traces of the light pulses passing through individual optical fibers, and the shaded region exemplifies the beam profile of a light pulse exiting an optical fiber. The inset is a photograph of the actual fiber bundle.

In this letter, we present a new time-multiplexing device that can be more easily manufactured to produce a high degree of time multiplexing without the concerns of light leakage between neighboring time-delay channels. Our device consists of a bundle of optical fibers of various lengths, wherein time multiplexing is created by the relative length differences among the fibers. To avoid the aforementioned light leakage, we aligned the ends of all the fibers at the input and output planes, while the length differences were compensated by slight bending of the fibers (Fig. 1). Because of the length differences in the fibers, input light pulses that simultaneously enter two fibers will exit the bundle with a temporal separation of

2 $${\rm{\Delta }}t=\frac{n{\rm{\Delta }}l}{c},$$

where *n* is the refractive index of the fiber core, Δ*l* is the length difference between the two fibers, and *c* is the speed of light in the vacuum. It was suggested that with a temporal separation twice the pulse duration *τ*, time multiplexing can be created between two foci^5^. Thus, the minimal length difference between any two fibers should be

3 $${\rm{\Delta }}{l}_{{\rm{\min }}}=\frac{2\tau \,c}{n}.$$

Δ*l*_*min*_ is estimated to be ~14 *μ*m in our setup, where we had *τ* ≈ 35 fs and *n* ≈ 1.5. Using this estimate, we developed a fiber bundle prototype consisting of 168 optical fibers (see *Methods* for details). To create a sufficient length variation, we separated the fibers into 84 groups, each of which contained two fibers that were meant to be cut into the same length, with a designed ~200 *μ*m length difference implemented between spatially adjacent groups. Meanwhile, the limited precision of our fiber-cutting method gave rise to a modest length fluctuation within each fiber pair, with a standard deviation ~30 *μ*m (Fig. S1). With Δ*l*_*min*_ ≈ 14 *μ*m and by combining the designated inter-group length differences and the stochastic intra-group length fluctuation, a statistical analysis (see *Methods* for details) showed that we could create ~146 unique time delays, nearly 50 times higher than what has ever been achieved in conventional time-multiplexing devices^7^. The fibers were then packed and butt-coupled with the ultrafast light pulses, with the ends of all the fibers being aligned in the same planes. Due to the cladding of fibers and the unused inter-fiber space in the packed fiber bundle, there was inevitable loss of light at the light-fiber coupling interface. One way to enhance the coupling efficiency is to apply high-precision alignment and assembly of a light-coupling microlens array to the fiber-array bundle. Here, for simplicity, we used large-core multimode fibers and directly butt-coupled the fiber bundle with the light. Such straightforward coupling provided a coupling efficiency of ~5%. Note that the primary purpose of using Eqns 2 and 3 was to estimate the minimal length difference within the fiber bundle, by which we can create a sufficient separation of arrival time. For such a purpose, it might not be mandatory to include the model dispersion effect. Nevertheless, the model dispersion effect can become significant in multimode fibers, leading to a pulse broadening effect, which we will address later.

**Figure 2:**
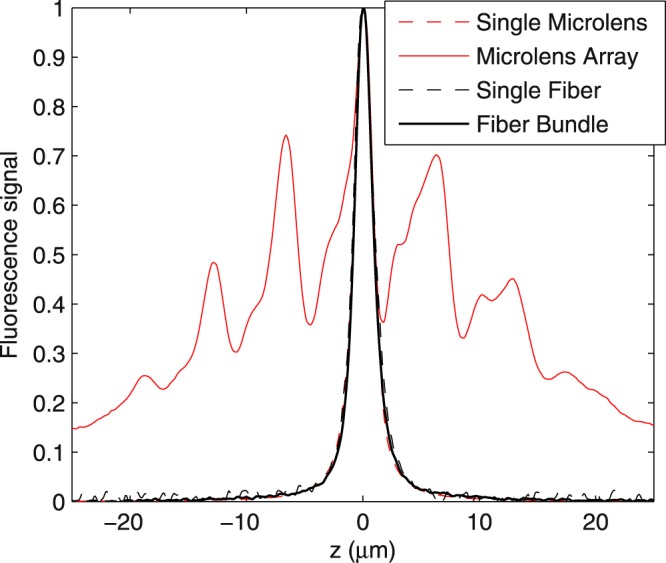
Comparison of axial response curves of multiphoton excitation with and without time multiplexing. The similarity of the axial responses of fiber-bundle (solid-black line) and single-fiber (broken-black line) illumination demonstrates that the length differences among the fibers can indeed create time multiplexing to prevent out-of-focus excitation, which is extensive in the non-time-multiplexed microlens-bundle illumination geometry (solid-red line). The inset shows the axial response curves near the focal plane of the objective lens.

Having the fiber bundle assembled, we examined if it can overcome one of the major issues in the non-time multiplexed wide-field multiphoton excitation system, namely, the interfocal interference, which arises as all the foci are excited at the same time in the non-time multiplexed system, leading to a significant out-of-focus background. To ascertain whether our fiber bundle-based time-multiplexed setup prevents interfocal interaction and suppresses out-of-focus excitation, we compared the axial response of our system with that of (1) a non-time-multiplexed multifocal system that has foci spacing similar to ours and (2) single-focus systems that, by design, have no interfocal interference. Here, system 1 represents conventional multifocal multiphoton microscopy that uses a microlens array for illumination^8^, while system 2 provides a quantitative comparison to conventional single-focus multiphoton microscopy^9^. The axial responses of each system were measured by imaging the same thin fluorescent layer at sequential distances from the focal plane of the microscope objective. For a fair comparison of out-of-focus excitation between our system and a non-time-multiplexed system, we used a microlens array to mimic the non-time-multiplexed system in that the full width at half maximum (FWHM) of the axial response from the aperture created by a single microlens (Fig. 2, red broken line) is similar to that from single-fiber illumination (see *Methods* for details). Remarkably, we found that the axial response of our fiber-bundle system resembles those from single-focus systems (Fig. 2), confirming that the fiber length differences in our system do produce sufficient temporal separations among the spatially neighboring foci, thereby preventing out-of-focus excitation.

As aforementioned, using multimode fibers can lead to a significant modal dispersion effect, as the axial response curve being a mixture of the axial responses from several optical modes, among which the higher-order modes exhibit longer focal depths that can broaden the mixed-mode axial response curve. Consistently, the single-fiber illumination showed an axial response curve with a FWHM ~1.8 *μ*m (Fig. 2, black broken line), more than the FWHM obtained from an optimized conventional single-focus multiphoton microscope using the same objective (FWHM ~0.9 *μ*m)^10^. To estimate such dispersion-induced pulse broadening effect, we simulated the group velocities in dominant modes (i.e., modes contributing to >99% of the total power measured at the output end of the fiber bundle), by which we computed the corresponding modal time delays. Briefly, the group velocity of 268 transverse modes were simulated using the commercial software OptiFiber (Optiwave Systems Inc.; details can be found in the Supplementary Information Section 2). The result indicated that the pulse was broadened by ~16 fs (through the variation of group velocities; see Supplementary Information and Fig. S2), which was ≈*τ*/2 of our light source; hence, the efficiency of two-photon fluorescence excitation was reduced by ~30%. Nevertheless, the result also showed a small standard deviation of dispersion parameters (0.24 ps/nm/km) among dominant modes (Fig. S2b), as compared to the absolute value of the average dispersion parameter (116.7 ps/nm/km), suggesting that the group delays within different modes can be compensated by a standard pulse compressor.

**Figure 3:**
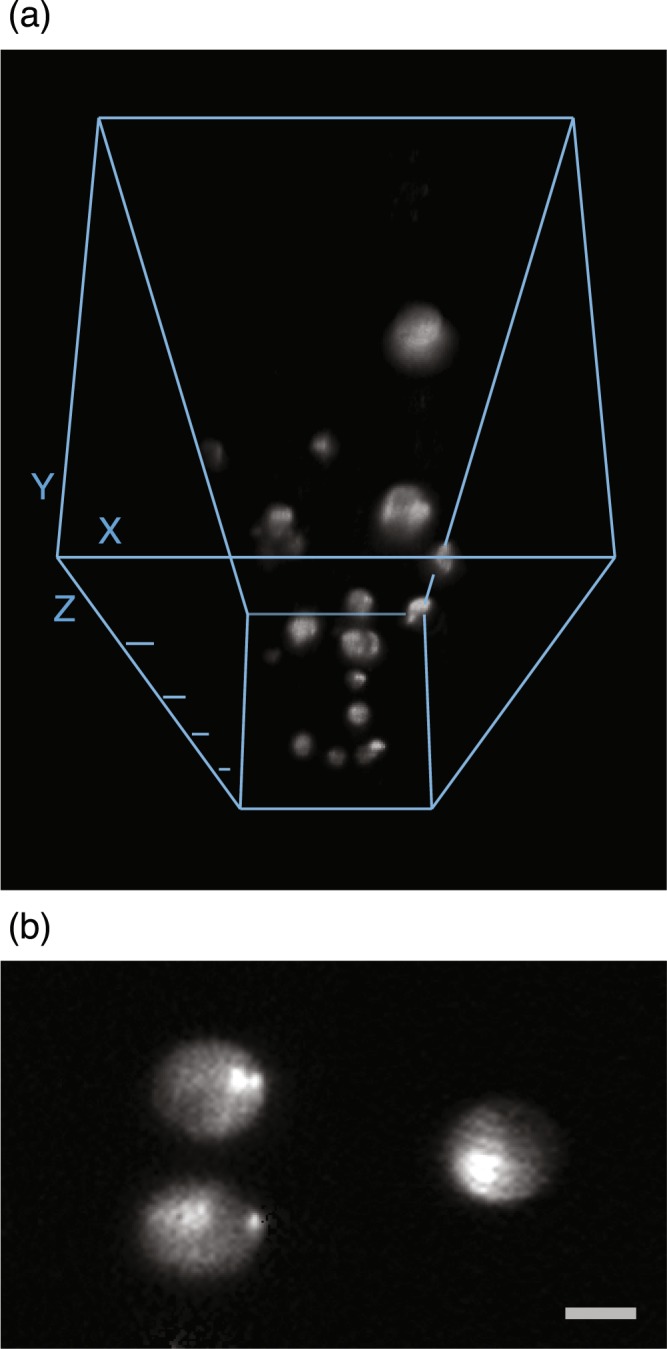
Three-dimensional reconstruction (**a**) and an optical section (**b**) of fluorescent microspheres embedded in agarose gel. We used the 3D Viewer of ImageJ to reconstruct the three-dimensional view from 332 sequential optical sections with a 3-*μ*m depth interval. For each optical section, we integrated four images of 1-ms exposure obtained by translating the fiber bundle to four different positions, equivalent to an overall frame rate of 250 fps. The depth difference between adjacent Z ticks in (**a**) is 200 *μ*m, and the scale bar in (**b**) is 10 *μ*m. The microspheres are 15 *μ*m in diameter.

Next, we evaluated the fast optical-sectioning capability of our system. To do so, we imaged fluorescent microspheres embedded in an agarose gel. For each optical section, we used four translational steps of the fiber bundle to homogenize the illumination field, with each step exposed for 1 millisecond, equivalent to an overall frame rate of 250 frames per second (fps). Figure 3 shows the three-dimensional visualization of the 15-*μ*m-diameter microspheres and one single section of the sample. Despite the limited field of view due to the small number of fibers used in our prototype and the slightly-degraded image quality due to the nonuniform fiber spacing and coupling efficiency (by our preliminary coupling method), we were able to conduct optical-sectioning imaging for a fluorescence-labeled biological sample (Supplementary Movie 1). The sectioning capability was further demonstrated by comparing the z-sectioning images of the microlens array-based non-time multiplexed setup and our fiber bundle-based time-multiplexed setup, where the out-of-focus background was evident in the microlens array-based setup but suppressed in the fiber bundle-based setup (Fig. S3). Together, these results demonstrated the high degree of time multiplexing achieved by the fiber-bundle method, which efficiently prevented out-of-focus excitation and in turn enabled fast three-dimensional optical sectioning.

Having shown the advantages of the proposed time multiplexing method, we next discuss its achievable degree of time multiplexing, limits of imaging speed and depth, and capacity for optical sectioning. We will also address how the observed uneven coupling efficiency may be avoided by using single-mode fibers and currently available fiber coupling techniques. First, neglecting the physical constraints and manufacturing difficulties associated with conventional time-multiplexing devices, in our system the degree of time multiplexing is limited primarily by relative group delays generated by fibers of different lengths (which lead to unequal excitation among the foci), and the achievable unique time delays (*N*_TM_) obey

4 $${N}_{{\rm{TM}}}\le (\frac{n}{2\,\alpha \,c\,|D|})\cdot \tau ,$$

where *D* is the dispersion parameter of the optical fiber, and *α* is the transformation constant of transform-limited light pulses (see *Methods* for details). In our system, *τ* ≈ 35 fs and *D* ≈ −117 ps/nm/km, which yield *N*_TM_ ~ 1,500, much larger than the aforementioned number of unique time delays required for scanningless imaging (~300).

Second, regarding the imaging speed, we should point out that the current setup is a proof-of-concept prototype. Our system is primarily limited by the instrumental parameters such as the repetition rate of the ultrafast pulse train, the frame rate of the camera, and the translation rate of the fiber bundle (only if translation is required for a homogeneous illumination, which is unnecessary if the bundles are made of closely spaced, low numerical-aperture fibers). These issues are commercially solvable as there are ultrafast oscillators to provide repetition rates up to 1–10 GHz at average powers >1W and regenerative amplifiers to produce pulses with energy larger than several mJ and repetition rates up to many tens of kHz (if larger single pulse energies are needed). Likewise, there are scientific-grade CMOS cameras with rates >1,000 fps (at ~100 × 100 pixel number) and mechanical stages with step rates typically of order of kHz for fast fiber-bundle translation. For even faster operation galvanometric mirrors that translate the foci at rates ≥50 kHz can be used, in an optical design similar to a previously demonstrated multifocal system^11^. Thus, regardless of the cost, it is possible to push the imaging speed of our technique beyond 1,000 fps. In addition to instrumental limitations, we should note that several factors in biological imaging applications can significantly limit achievable imaging speeds, such as signal-to-noise ratio of the images, photobleaching, and heat absorptions induced by the near-infrared illumination. The significance of these factors can vary greatly from one imaging application to another with a strong dependence on biological specimens, and therefore require further studies to clarify.

Third, regarding the image depth, our system differs from conventional single-focus scanning multiphoton microscopy in that blurring in our system is primarily due to optical scattering and aberration within the sample. This issue also occurs in SPIM and structured illumination microscopy (SIM), because both use camera sensor arrays to collect emission signals. If the fluorescence signal is strong enough, the scattering-induced blur can be numerically removed by using the foci patterns to generate structured illumination effect^4^, which can also enhance the axial resolution^6,12,13^. Alternatively, one can apply spatial registration of the signal, i.e., assigning the signal collected by certain pixels of the sensor array to individual foci, and use the spatial information associated with the foci to reconstruct the entire image^11^. Likewise, one can use frequency registration, i.e., encoding the amplitudes of foci with various frequencies to reconstruct the image through frequency analysis of the collected fluorescence signals^14^. We should note, however, that spatial registration cannot be applied to high-frame-rate scanningless microscopy, because it requires certain sparsity of the foci spacing (for spatial registration of individual foci), which in turn could compromise the imaging speed.

Fourth, regarding the optical sectioning capability, our system can achieve the same level as conventional single-focus multiphoton microscopy, provided that it is fully optimized. Such optimization requires collimating the light (from the fiber to the microscope objective) with a flat wavefront and amplitude distribution. Thus, using bundles of single-mode fibers is highly preferred. To enhance coupling efficiency, a high-precision alignment and assembly of a light-coupling microlens array with the fiber-array bundle is then required, due to the relatively small core size of single-mode fibers. Fortunately, technology for this highly demanding work has recently become commercially available, due to the fast-growing demands of highly parallel optical communication^15,16^. Such technology might provide not only a coupling efficiency >90%, but also a potential way to resolve the uneven coupling efficiency observed in our prototype fiber bundle.

In summary, we propose and demonstrate a simple method to provide the highest degree of time multiplexing ever achieved in multifocal multiphoton microscopy. By introducing length differences within a bundle of optical fibers, we create a time multiplexing effect that efficiently prevents out-of-focus excitation even in densely packed foci. Using simple far-field optical imaging, the maximal imaging speed in our current setup can reach 250 fps, with a lateral resolution primarily limited by far-field diffraction, which depends on the wavelength of the emitted photon and the numerical aperture of the objective. Due to the use of multimode fibers, however, the axial resolution in our current setup (~1.8 *μ*m) is worse than an optimized single-focus multiphoton microscope (~0.9 *μ*m). Likewise, the small number of optical fibers limits our field of view (~1/30 of the camera sensor area in this study). Nevertheless, these issues can be resolved by commercially available devices and technologies, e.g., increasing the field of view by elaborated fiber assembly technology. Once optimized, the proposed method will surpass currently available wide-field optical-sectioning fluorescence microscopies in terms of imaging speed and system simplicity to achieve spatial resolutions equivalent to conventional single-focus multiphoton microscopy. With an imaging speed potentially beyond 1,000 fps, we believe that our technique will provide a powerful imaging tool in future life science research.

## Methods

### Fiber bundle manufacturing

As described in the main text, we assembled the fiber bundle with large-core multimode fibers (core diameter ≈62.5 *μ*m and cladding diameter ≈125 *μ*m, dispersion parameter ≈−116.7 ps/nm/km at 800 nm, YOFC^®^) of various lengths. To cut the optical fibers into designated lengths, we fixed fibers on a precision translational stage to adjust the lengths, and cut the fibers with a cleaver next to the stage. When assembling the fibers, both ends of the bundle were aligned perpendicular to the optical axis, as shown in Fig. 1. The length differences were compensated by slightly bending the fibers–the optical bending loss is negligible due to the relatively short length differences (Δ*l*_max_ ≈ 17 mm) compared with the average length of the fiber bundle (≈200 mm). After assembly, the physical strength of the fiber bundle was further enhanced by applying UV-cured epoxy near the ends of the fibers, followed by polishing and ultrasonic cleaning at both ends of the fiber bundle.

### Statistical analysis of the degree of time multiplexing

To estimate the degree of time multiplexing created by our fiber preparation, we numerically simulated the length distribution of the resulting fiber bundle. As shown in Supplementary Fig. 1, our fiber cutting method produced a Gaussian-like length distribution with a standard deviation of ~30-*μ*m. To include such stochasticity, we added a Gaussian random variable with a mean at 0 *μ*m and a standard deviation of 30 *μ*m to the lengths of the fibers in each length group. We then compared the length differences among all 168 fibers in the bundle and determined the number of unique time delays. Specifically, we reduced 168 by the number of fibers that had a length difference less than Δ*l*_min_ with another fiber in the bundle to obtain the number of unique time delays (Δ*l*_min_ indicates the minimal length difference for creating two unique time delays, set to be 14 *μ*m in the simulation). By averaging 1,000 simulations, we concluded that ~146 ± 4 unique time delays can be produced by our fiber bundle preparation.

### Development of the optical system

The light source of our system is a Ti:Sapphire ultrafast regenerative amplifier (Legend Elite-USP-1k-HE, Coherent, Inc.) seeded with an ultrafast oscillator (Mantis-5, Coherent, Inc.), and is butt-coupled into the fiber bundle. The repetition rate and pulse duration of the ultrafast pulse train are ~1 kHz and ~35 fs, respectively, and the central wavelength is ~800 nm. In our infinity-corrected optical setup (Fig. 1), the output end of the fiber bundle was placed at the focal plane of the collimation lens (*f* = 150 mm plane-convex lens, KPX100AR.16, Newport Corp.). The temporally and spatially separated pulses entered an inverted microscope frame (IX71, Olympus) through the back port, and were reflected upward to the microscope objectives by a beamsplitter (20RQ00UB.2 with customized dimensions, Newport Corp.). The emitted fluorescence then formed an image on the sensor array of an electron-multiplying CCD camera (iXon DU-885K, Andor). To measure the axial response shown in Fig. 2, we used a high numerical aperture oil-immersion lens (PlanApo N 60X NA 1.42, Olympus) equipped with a high-precision piezo stage (P-725 PIFOC^®^, Physik Instrumente) for axial translation. The objective used for acquiring the optical sections shown in Fig. 3 and Supplementary Movie 1 is a long-working-distance water-immersion lens (XLUMPlanFL N 20X NA 1.00, Olympus). We used a DC-motor XY stage (MS-2000, Applied Scientific Instrumentation) to laterally translate the fiber bundle.

### Measurement of axial responses

We measured the axial response by imaging a thin fluorescent layer with the microscope objective translated through 200 sequential depths at 0.3-*μ*m intervals. The thin layer was made by sandwiching a tiny drop of a fluorescent dye between a #1.5 coverslip and a non-fluorescent quartz microscope slide (Ted Pella, Inc.). The fluorescent dye was diluted 3 times from a saturated 1, 8-ANS solution of dimethylformamide. The thickness of the thin layer was estimated to be ≤1 *μ*m by dividing the volume of the dye drop with the coverslip area. To measure the axial response of the non-time-multiplexing microlens array, we replaced the fiber bundle with a square-microlens array (lens pitch ≈100 *μ*m, focal length ≈3 mm, Flexible Optical B.V.) that formed foci spacing similar to our fiber bundle. To have a similar number of foci as those generated by our fiber bundle, we placed a mechanical iris in front of the microlens array to partially block the excitation beam.

We conducted single-fiber illumination by placing a 50 *μ*m pinhole (P50S, Thorlabs, Inc.) next to the input plane of the fiber bundle to selectively couple the excitation beam to a single fiber. Because of the geometry of the mechanical mount and the high filling factor of the microlens array, this pinhole-based technique required modest corrections to properly model a single-microlens illumination geometry. To obtain the proper axial response curve equivalent to that of single-microlens illumination, we first applied geometric optics calculations to estimate the equivalent aperture formed by a single microlens, and then measured the axial response through a conventional single-focus multiphoton microscope with the equivalent aperture placed in front of the back aperture of the microscope objective. Each axial response curve was averaged from 15 separate z-scans.

### Estimating the upper bound of the number of unique time delays

As described in the main text, the degree of time multiplexing of our technique is limited by the relative group velocity dispersion of the ultrafast pulses among the fibers. The relative group delays generated by different length fibers results in pulse duration variations in the exiting light pulses, which can lead to unequal excitation among the foci. To mitigate this effect, the overall group delay Δ*τ*_GVD_ should be restricted such that

5 $${\rm{\Delta }}{\tau }_{{\rm{GVD}}}\approx {\rm{\Delta }}{l}_{{\rm{\max }}}\cdot {\rm{\Delta }}\lambda \cdot |D|\le \tau ,$$

where Δ*l*_max_ is the maximum of fiber length difference in the bundle, Δ*λ* is the spectral span of a transform-limited light pulse, and *D* is the dispersion parameter of the optical fiber. With the constraint of Eqn. 5, the brightest foci will be no more than twice as bright as the darkest ones. Given *τ* ≈ *α*/Δ*λ* for transform-limited light pulses (*α* is a transformation constant), Eqs 3 and 5 suggest that the largest number of unique time delays is bounded by

6 $${N}_{{\rm{TM}}}\approx \frac{{\rm{\Delta }}{l}_{{\rm{\max }}}}{{\rm{\Delta }}{l}_{{\rm{\min }}}}\le (\frac{n}{2\,\alpha \,c\,|D|})\cdot \tau .$$

For commonly used near-infrared optical fibers and transform-limited Gaussian pulses, $\frac{n}{2\alpha c|D|}$ is ~50 fs^−1^ at *λ*_0_ ≈ 800 nm. In our system where *τ* ≈ 35 fs and *D* ≈ −116 ps/nm/km, we obtained that *N*_TM_ is upper-bounded by ~1,500.

## Electronic supplementary material

Movie S2

Movie S1

Supplementary Information

LaTeX Supplementary File
