# Supplementary material for: Fiber-bundle illumination: realizing high-degree time-multiplexed multifocal multiphoton microscopy with simplicity
Source: Sci Rep. 2018 Oct 5;8:14863. doi: 10.1038/s41598-018-33286-1 (PMC6173774; doi:10.1038/s41598-018-33286-1)
Supplement: Supplementary file 3 — Supplementary Information [file 41598_2018_33286_MOESM3_ESM.pdf]

Supplementary Information for *'Fiber-bundle illumination: realizing high-degree time-multiplexed multifocal multiphoton microscopy with simplicity'*

Jiun-Yann Yu,[1,6,*] Sunduck Kim,[2,*] Young Bo Shim,[2,*] Daniel B. Holland,[3] Marco A. Allodi,[3] Chao-Yuan Yeh,[4], Geoffrey A. Blake,[3,5] Young-Geun Han,[2] & Chin-Lin Guo[6]

[1]*Division of Biology and Biological Engineering, California Institute of Technology, Pasadena, CA 91125, USA*

[2]*Department of Physics, Hanyang University, Seoul 133-791, South Korea*

[3]*Division of Chemistry and Chemical Engineering, California Institute of Technology, Pasadena, CA 91125, USA*

[4]*Department of Pathology, University of Southern California, Los Angeles, CA 90033, USA*

[5]*Division of Geological and Planetary Sciences, California Institute of Technology, Pasadena, CA 91125, USA*

[6]*Institute of Physics, Academia Sinica, NanKang District, Taipei, Taiwan 11579, ROC*

[*]*These authors contributed equally to this work.*

1 Statistical analysis of lengths of optical fibers

To quantify the stochasticity in fiber lengths due to our fiber cutting procedure, we measured the lengths of 160 fibers that were prepared through the same procedure, and plot the histogram of the differences between the measured lengths and the designated length. The distribution of length

differences is in a good agreement with a Gaussian distribution with a standard deviation of 30 μm (blue line in Supplement Fig. 1).

2 Estimation of pulse broadening caused by modal dispersion in a multimode fiber

Because different transverse modes travel at different group velocities in a multimode fiber, inter-mode time delays can occur after the ultrafast pulses travel a certain distance in the fiber. If l is the length of the traveling distance, n is the refactory index, and v is the group velocity, we can estimate the traveling time τ of various modes using the relation $\tau = l/nv$. From the distribution of the traveling time, we can then obtain the relative time delays, by which we estimate the pulse broadening effect. To do so, we simulated the group velocity of 268 transverse modes of the fiber, using a commercial software package (OptiFiber, Optiwave Systems Inc.). For an intuitive visualization, we converted the result into a power-group velocity distribution (Supplementary Fig. 2(a)), which has a standard deviation $\sim 3 \times 10^3$ m/s. Such a standard deviation, given the length of our fibers to be $\sim$ 0.2 m, results in a standard deviation of the relative time delays $\sim$ 16 fs.

We noted that the difference in group velocity dispersions among different transverse modes may lead to pulse broadening that cannot be compensated trivially. As such, we simulated the dispersion parameters of the dominant modes, by which we estimated the pulse broadening effect. The result shows a small standard deviation of dispersion parameters ($\sim$ 0.24 ps/nm/km) among dominant modes (Supplementary Fig. 2(b)), as compared to the absolute value of the average dispersion parameter ($\sim$ 116.7 ps/nm/km), suggesting that the group delays within different modes

can be compensated by a standard pulse compressor.

3 Optical-sectioning images of a fluorescence-labeled chicken skin tissue

To examine the feasibility of our technique for biological imaging applications, we conducted an optical-sectioning imaging on a fluorescence-labeled biological sample (Supplementary Movie 1). The sample was a dorsal skin tissue of a day-8 chicken embryo fixed in methanol/DMSO (in a 4:1 ratio), stained with 0.1% propidium iodide, and mounted in benzyl alcohol/benzyl benzoate (in a 1:2 ratio). Each optical section consists of 12 lateral translation steps of the fiber bundle, and the exposure time for each step is 20 millisecond. The unequal spacing and coupling efficiency among individual fibers resulted in the bright and dark stripes observed in Supplementary Movie 1. The low signal-to-noise ratio images are mainly due to the mismatch of the available laser wavelengths ($\sim$ 800 nm) and the optimal two-photon excitation wavelength of propidium iodide (> 1000 nm). Nevertheless, the signal is expected to be 10 times stronger if appropriate laser wavelengths were used. Supplementary Figure 3 shows the intensity profiles of the illumination field, by imaging a thin fluorescent layer, at the 12 translation steps used to acquire Supplementary Movie 1.

Note that the sample used in Supplementary Movie 1 did not show any specific tissue architecture. For well-developed or differentiated biological tissues, the structures are usually visualized by applying multi-color staining and multi-channel imagining on various molecules (e.g., nucleus, Golgi network, intracellular signaling molecules, cytoskeleton, and extracellular matrix). Unfortunately, our setup is a proof-of-concept prototype that can only be used for single channel imagining.

As such, we did not choose well-developed samples for the demonstration. The day 6-8 dorsal skin tissues from chicken embryos were from our collaborator (Prof. Cheng-Ming Chuong, Pathology Department, Keck School of Medicine, University of Southern California) who studies how dermal condensation and placode formation spontaneously occurs from initially homogeneous conditions. In other words, we did not expect that such early-staged samples would exhibit any specific tissue architectures. Nevertheless, they are good samples for us to test the capability of our prototype setup in the long-term live tissue imaging.

We have also compared the optical sectioning effect between our fiber bundle-based time-multiplexed setup and the microlens array-based non-time multiplexed setup. In a non-time multiplexed system using the microlens array, the interfocal interference among neighboring focal fields is not eliminated and can significantly reinforce the defocused field, leading to an inevitable out-of-focus background, which is attenuated in our fiber bundle-based time-multiplexed setup (Fig. S3). Specifically, Supplementary Figure 3 (a) and (b) show the z-sectioning images obtained by the microlens array and the fiber bundle, respectively, at various z positions above or below a thin fluorescent layer. Supplementary Figure 3 (c) shows the corresponding orthogonal view of z-sectioning profiles, by which we can see an evident out-of-focus background in the microlens array-based setup, which was suppressed in our fiber bundle-based setup.

Supplementary Movie 1: A dorsal skin tissue of a day-8 chicken embryo fixed in methanol/DMSO (in a 4:1 ratio), stained with 0.1% propidium iodide, mounted in benzyl alcohol/benzyl benzoate (in a 1:2 ratio), and imaged by our setup with 12 translation steps.

Supplementary Movie 2: The corresponding z-stack movie for Fig. S3.

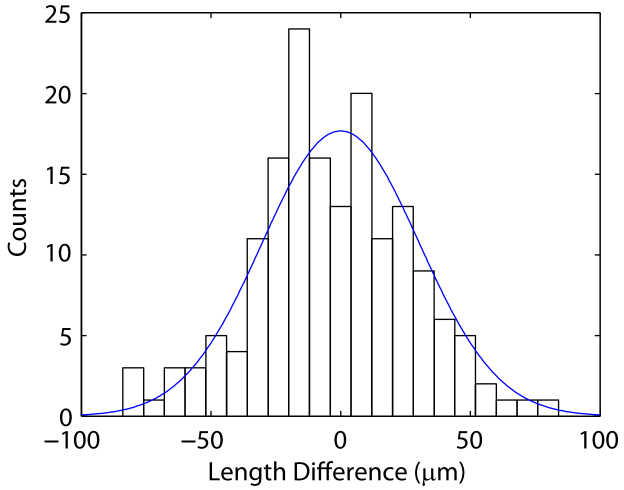
Counts
Length Difference (μm)
0
5
10
15
20
25
−100
−50
0
50
100

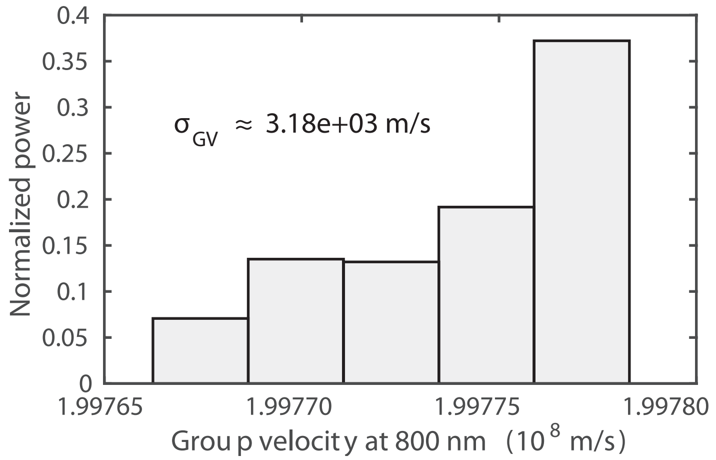
0.4
0.35
0.3
0.25
0.2
0.15
0.1
0.05
0
Normalized power
σGV ≈ 3.18e+03 m/s
1.99765
1.99770
1.99775
1.99780
Grou p velocit y at 800 nm (10 8 m/s)

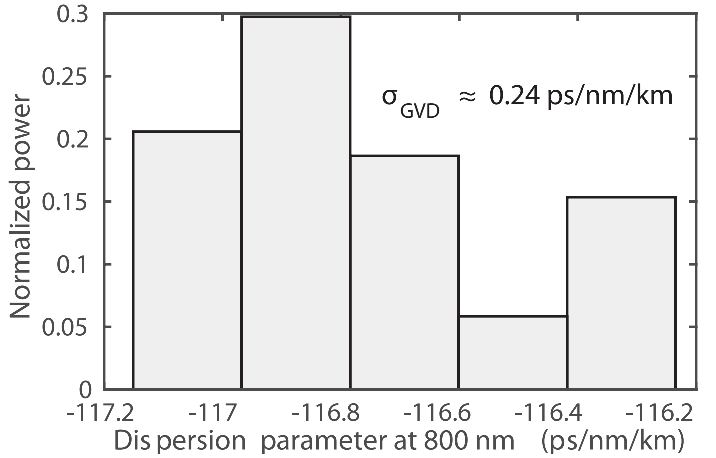
0.3
0.25
0.2
0.15
0.1
0.05
0
Normalized power
σGVD ≈ 0.24 ps/nm/km
-117.2
-117
-116.8
-116.6
-116.4
-116.2
Dis persion parameter at 800 nm (ps/nm/km)

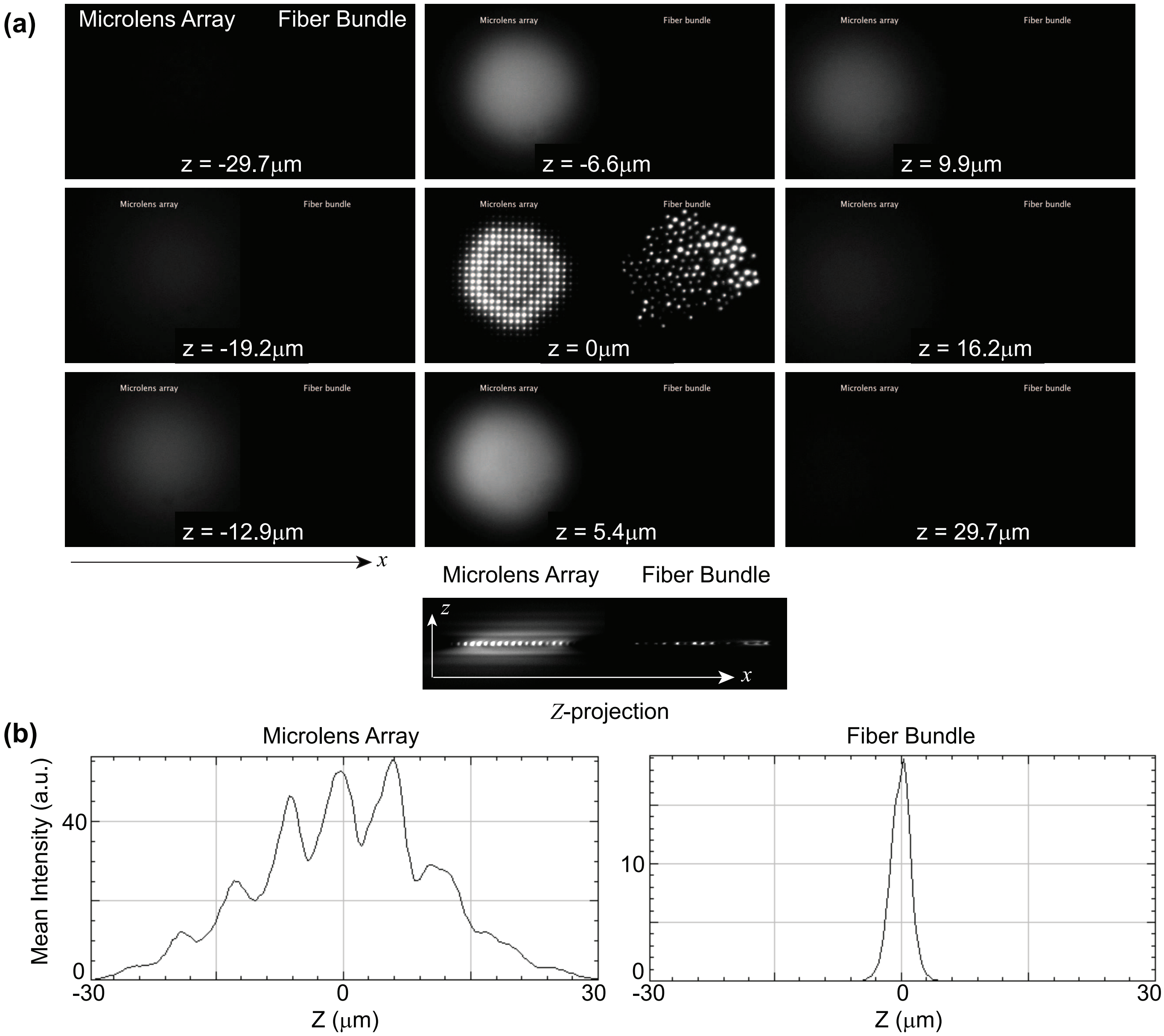
(a)
Microlens Array
Fiber Bundle
z = -29.7μm
z = -6.6μm
z = 9.9μm
z = -19.2μm
z = 0μm
z = 16.2μm
z = -12.9μm
z = 5.4μm
z = 29.7μm
x
Microlens Array
Fiber Bundle
z
x
Z-projection
(b)
Microlens Array
Fiber Bundle
Mean Intensity (a.u.)
40
0
10
0
-30
0
30
-30
0
30
Z (μm)
Z (μm)

Figure 1 Length distribution of fibers that were meant to be cut into the same length. The blue line indicates a Gaussian distribution of a 30μm standard deviation.

Figure 2 Statistical analysis of (a) group velocity and (b) group velocity dispersion in the multimode fiber used in our setup. σ_{GV} and σ_{GVD} denote the standard deviation of group velocity and group velocity dispersion, respectively.

Figure 3 (a) The z-sectioning images obtained by illumination using a microlens array or a fiber bundle at various z positions above or below a thin fluorescent layer. The corresponding orthogonal view of z profiles is shown below. (b) The profile of average intensity for each z-sectioning image taken in (a).
